# From Type 2 Inflammation to Mucociliary Dysfunction: Individualized Pulmonary Rehabilitation in Children with Asthma and Bronchiectasis

**DOI:** 10.3390/children13101430

**Published:** 2026-10-18

**Authors:** Anca Vidrighin, Iulius Jugănaru, Valentin-Cristian Iovin, Cosmina Diaconu, Andreea-Adriana Neamțu, Cristian Sarău, Diana Luisa Lighezan, Cristinel Gabriel Diaconu, Corina Porr

**Affiliations:** 1 Department of Paediatrics, Faculty of Medicine, Lucian Blaga University of Sibiu, 550169 Sibiu, Romania; 2 Department XI of Pediatrics, Discipline I of Pediatrics, “Victor Babeș” University of Medicine and Pharmacy, 300041 Timișoara, Romania; 3 Department of Pediatrics I, “Louis Țurcanu” Children’s Emergency Hospital, 300011 Timișoara, Romania; 4 Doctoral School Department, “Victor Babeș” University of Medicine and Pharmacy, 300041 Timișoara, Romania; 5 Department of Nursing and Dental Medicine, Faculty of Medicine, Lucian Blaga University of Sibiu, 550169 Sibiu, Romania; 6 Clinic of Physical Medicine and Rehabilitation, County Clinical Emergency Hospital Sibiu, 550245 Sibiu, Romania; 7 Department of Toxicology, “Victor Babeș” University of Medicine and Pharmacy, 300041 Timișoara, Romania; andreea.neamtu@umft.ro; 8 Research Centre for Pharmaco-Toxicological Evaluation, “Victor Babeș” University of Medicine and Pharmacy, 300041 Timișoara, Romania; 9 Department of Pathology, Clinical County Emergency Hospital of Arad, 310037 Arad, Romania; 10 Department of Pathology, “Pius Brînzeu” Clinical County Emergency Hospital, 300723 Timișoara, Romania; 11 Medical Semiology I, Department V Internal Medicine I, “Victor Babeș” University of Medicine and Pharmacy, 300041 Timișoara, Romania; 12 Department of Hematology, “Victor Babeș” University of Medicine and Pharmacy, Eftimie Murgu Square, No. 2, 300041 Timișoara, Romania; 13 Department of Physiology, Faculty of Medicine, Lucian Blaga University of Sibiu, Lucian Blaga Street 2A, 550169 Sibiu, Romania; gabriel.diaconu@ulbsibiu.ro; 14 Department of Radiology, Pneumology and Tuberculosis Hospital, 550196 Sibiu, Romania; 15 Allergology Department, Faculty of Medicine, Lucian Blaga University of Sibiu, 550169 Sibiu, Romania; 16 Allergology Department, County Clinical Emergency Hospital Sibiu, Bulevardul Corneliu Coposu 2–4, 550245 Sibiu, Romania

**Keywords:** pediatric asthma, bronchiectasis, airway clearance, mucociliary dysfunction, Type 2 inflammation, exercise training, exercise-induced bronchoconstriction, respiratory muscle training, pulmonary rehabilitation

## Abstract

**Highlights:**

**What are the main findings?**

- Type 2 inflammation, mucus hypersecretion, impaired mucociliary clearance, infection, and structural airway injury may interact in children with coexisting asthma and bronchiectasis.
- Pulmonary rehabilitation should be individualized according to the predominant clinical and functional limitations, including mucus retention, bronchial hyperresponsiveness, exercise intolerance, respiratory muscle dysfunction, and deconditioning.

**What is the implication of the main finding?**

- A mechanism-informed rehabilitation approach may help integrate airway-clearance techniques, exercise training, asthma control, infection management, and functional reassessment within multidisciplinary care.
- Prospective pediatric studies are needed to validate individualized rehabilitation strategies and determine their impact on clinically meaningful and patient-centered outcomes.

**Abstract:**

Asthma and bronchiectasis may coexist in children, particularly in those with severe or difficult-to-treat respiratory disease, creating a complex clinical setting in which airway inflammation, bronchial hyperresponsiveness, mucus retention, impaired mucociliary clearance, recurrent infection, and functional impairment interact. This review critically examines the interacting pathophysiological mechanisms involving Type 2 airway inflammation, mucus hypersecretion and plugging, mucociliary dysfunction, infection, and structural airway injury in children with coexisting asthma and bronchiectasis. Particular attention is given to the functional consequences of this interaction, including exercise intolerance, physical deconditioning, respiratory muscle dysfunction, and reduced participation in daily life and age-appropriate physical activity. Available evidence on airway-clearance techniques, structured exercise training, respiratory muscle training, and the management of exercise-induced bronchoconstriction is critically evaluated, with pediatric evidence clearly distinguished from adult-derived extrapolations. We propose a mechanism-informed rehabilitation framework in which interventions are tailored to the predominant clinical and functional features of each child, including mucus retention, bronchial hyperresponsiveness, reduced exercise capacity, respiratory muscle impairment, and functional decline associated with recurrent exacerbations. This multidisciplinary approach integrates airway-clearance techniques, individualized exercise training, optimized asthma management, infection control, and longitudinal functional reassessment. Evidence specifically addressing children with coexisting asthma and bronchiectasis remains limited, and prospective pediatric studies are required to validate individualized rehabilitation strategies and determine their impact on long-term respiratory and patient-centered outcomes.

## 1. Introduction

Asthma and bronchiectasis are chronic respiratory disorders that may coexist in children, particularly in those with severe, persistent, or difficult-to-treat disease. Asthma is characterized by variable airflow limitation, airway hyperresponsiveness, and heterogeneous inflammatory pathways, whereas bronchiectasis is associated with abnormal bronchial dilatation, impaired mucociliary clearance, mucus retention, recurrent infection, and chronic airway inflammation [1,2,3]. When both conditions coexist, symptoms such as cough, wheeze, exertional dyspnea, recurrent exacerbations, and exercise intolerance may arise from overlapping mechanisms rather than from a single disease process [4,5,6,7,8,9].

The relationship between asthma and bronchiectasis in childhood remains incompletely understood. Although asthma–bronchiectasis overlap is increasingly recognized in adults, pediatric evidence remains limited, and coexistence should not automatically be interpreted as a distinct, well-defined clinical phenotype [4,5,6,7,8,9]. Nevertheless, structural airway abnormalities, including bronchiectatic changes, have been reported in children with severe asthma, supporting further investigation into the interplay between inflammatory airway disease and structural lung injury [10,11].

Several mechanisms may contribute to this association, including Type 2/eosinophilic airway inflammation, mucus hypersecretion and plugging, bronchial obstruction, impaired mucociliary clearance, microbial persistence, and neutrophilic inflammation [12,13,14,15,16,17,18,19,20]. These processes may interact bidirectionally, contributing to persistent respiratory symptoms, recurrent exacerbations, and progressive functional impairment [14,15,21,22].

Beyond conventional measures of disease control, affected children may experience airway secretion retention, exercise-induced bronchoconstriction, reduced exercise capacity, respiratory muscle dysfunction, physical deconditioning, and reduced participation in daily activities and physical activity [23,24,25,26,27,28,29,30,31]. These limitations may persist even when conventional clinical or spirometric measures appear relatively stable, indicating that functional outcomes should be considered alongside inflammatory and infectious disease control [23,24,25,32].

Pulmonary rehabilitation may therefore play an important role in comprehensive management. Airway-clearance techniques, structured exercise training, physical reconditioning, and respiratory muscle training are increasingly incorporated into the management of pediatric chronic respiratory diseases [1,24,25,26,27,28,29,30,31,33,34,35,36,37,38,39,40,41,42]. However, evidence specifically addressing children with coexisting asthma and bronchiectasis remains fragmented. Concomitant asthma may also influence the tolerability and implementation of airway-clearance techniques and exercise interventions, particularly in the presence of bronchial hyperresponsiveness and exercise-induced bronchoconstriction [3,29,30,31,36].

This review examines the potential mechanistic continuum linking Type 2 airway inflammation, mucus dysfunction, impaired mucociliary clearance, infection, and structural airway injury in children with coexisting asthma and bronchiectasis, with particular emphasis on pulmonary rehabilitation. We distinguish pediatric-specific evidence from evidence extrapolated from adult populations and propose a mechanism-informed framework for tailoring rehabilitation strategies to the predominant clinical and functional limitations of each child.

## 2. Literature Search Strategy and Review Approach

This review was designed as a structured state-of-the-art narrative review focused on the mechanistic, clinical, and rehabilitative implications of coexisting asthma and bronchiectasis in children.

PubMed/MEDLINE, Scopus, and Web of Science were searched for relevant publications available up to August 2026. Search terms included pediatric age groups, asthma, bronchiectasis, Type 2 inflammation, eosinophilic inflammation, mucus hypersecretion and plugging, mucociliary dysfunction, airway-clearance techniques, physiotherapy, pulmonary rehabilitation, respiratory rehabilitation, exercise, exercise-induced bronchoconstriction, and respiratory muscle training.

A representative PubMed search framework was:

(child* OR pediatric* OR paediatric* OR adolescent*) AND asthma AND (bronchiectasis OR bronchiectases) AND (airway clearance OR physiotherapy OR respiratory rehabilitation OR pulmonary rehabilitation OR exercise OR physical training OR mucociliary clearance).

Because the literature relevant to this review spans distinct clinical and mechanistic domains, complementary searches were also performed separately for pediatric asthma, pediatric bronchiectasis, and adult bronchiectasis rehabilitation. These searches combined population-specific terms with domain-specific concepts, including airway clearance, exercise training, respiratory muscle training, exercise-induced bronchoconstriction, mucus dysfunction, Type 2 inflammation, infection, and functional outcomes. Adult studies were retrieved through separate searches when pediatric evidence was insufficient for a specific mechanistic or rehabilitation question. The final literature search was completed in August 2026. No formal language restriction was applied; however, the literature included in the final synthesis was predominantly published in English.

Priority was given to original pediatric studies, including interventional and observational studies, as well as systematic reviews, meta-analyses, clinical practice guidelines, and expert consensus documents. Adult studies were included selectively when pediatric evidence was insufficient and when they provided relevant mechanistic, clinical, or therapeutic information. Adult-derived evidence was interpreted cautiously and was not considered direct evidence of pediatric effectiveness.

To further clarify the level and applicability of the evidence, pediatric studies were considered the primary basis for clinical and rehabilitation-related statements whenever available. Adult-derived evidence was used only to address specific mechanistic or therapeutic aspects for which pediatric data were limited or unavailable and was not regarded as directly equivalent to pediatric evidence. Throughout the review, pediatric evidence is distinguished from adult-derived extrapolations whenever this distinction is relevant to interpretation or clinical applicability. Given the narrative nature of the review and the heterogeneity of the available literature, no formal grading system for the level of evidence was applied; instead, interpretation took into account study design, consistency of findings, clinical relevance, and applicability to pediatric populations.

Titles and abstracts were screened for relevance to the predefined scope of the review, followed by full-text assessment of potentially eligible articles. Study selection was performed independently by two authors, who screened titles and abstracts and subsequently assessed potentially eligible full-text articles. Any disagreements were resolved by discussion and consensus. The reference lists of key reviews, clinical practice guidelines, expert consensus documents, and relevant original studies were also examined to identify additional publications of potential relevance.

Studies focusing exclusively on cystic fibrosis-associated bronchiectasis were excluded from the principal synthesis because of the distinct pathophysiological mechanisms and disease-specific management associated with cystic fibrosis.

Because the aim of this review was to integrate mechanistic, clinical, and rehabilitative evidence rather than to estimate a pooled treatment effect, no formal meta-analysis was performed. The evidence was synthesized across the following domains: asthma–bronchiectasis coexistence, inflammatory mechanisms, mucus and mucociliary dysfunction, infection and exacerbations, functional consequences, airway-clearance techniques, exercise training, respiratory muscle training, and exercise-induced bronchoconstriction.

The proposed pulmonary rehabilitation framework was developed on the basis of this evidence synthesis and should be regarded as an evidence-informed conceptual model rather than a prospectively validated pediatric treatment guideline.

## 3. Asthma and Bronchiectasis in Children: Coexistence, Association, or Distinct Phenotype?

Asthma and bronchiectasis may coexist in children, particularly in those with severe, persistent, or difficult-to-treat respiratory disease. However, unlike in adults, in whom asthma–bronchiectasis overlap is increasingly recognized as a clinically relevant subgroup, pediatric evidence remains limited, and coexistence should not automatically be interpreted as a distinct or validated clinical phenotype [4,5,6,7,8,9].

Pediatric bronchiectasis is characterized by persistent or recurrent respiratory symptoms, particularly chronic wet or productive cough, together with radiologically confirmed abnormal bronchial dilatation [1,2,33]. Importantly, bronchiectasis in childhood should not necessarily be regarded as irreversible, because early diagnosis and appropriate treatment may stabilize disease and, in selected patients, result in partial or even complete radiological improvement [1,2,43,44].

Asthma may contribute to mechanisms associated with bronchiectatic airway injury, including recurrent airway inflammation, mucus hypersecretion and plugging, airflow obstruction, and repeated exacerbations. Conversely, established bronchiectasis may aggravate asthma-related symptoms and disease burden through secretion retention, recurrent infection, and neutrophilic inflammation. The interaction may therefore be bidirectional, although a direct causal relationship between asthma and bronchiectasis in children has not been established [5,9,14].

Importantly, bronchiectasis should not be regarded as a direct consequence of asthma itself. In some children with asthma, recurrent or persistent lower-airway infections, potentially facilitated by underlying disorders such as immunodeficiency, primary ciliary dyskinesia, aspiration, or other predisposing conditions, may contribute more directly to the development and persistence of bronchiectatic airway damage. Thus, the coexistence of asthma and bronchiectasis should not be interpreted as a distinct asthma phenotype, but rather as the interaction of inflammatory airway disease with infectious, structural, and host-related factors.

Pediatric imaging studies support a clinically relevant association between severe asthma and structural airway abnormalities. Bronchiectatic changes and other structural alterations have been reported in selected children with severe asthma; however, these findings do not demonstrate that asthma itself is sufficient to cause bronchiectasis [10,11]. The principal pediatric studies and clinical guidance informing this association are summarized in Table 1.

The diagnosis of bronchiectasis in a child with asthma should prompt evaluation for potential underlying causes, including previous severe respiratory infection, immunodeficiency, primary ciliary dyskinesia, aspiration, congenital airway abnormalities, and cystic fibrosis. When clinically indicated, sweat chloride testing should be included as part of the etiological assessment, in accordance with pediatric bronchiectasis guidance. Conversely, wheeze and airflow obstruction in a child with bronchiectasis should not automatically be attributed to asthma. Whenever feasible, objective evidence of variable airflow limitation, bronchodilator responsiveness, and/or airway hyperresponsiveness should be sought, together with assessment of Type 2 inflammatory biomarkers when clinically appropriate [1,2,3,33,38,45].

In children with difficult-to-treat asthma, particularly when bronchiectasis, recurrent pulmonary infiltrates, marked eosinophilia, very high total IgE, or mucus plugging are present, sensitization to *Aspergillus fumigatus* and allergic bronchopulmonary aspergillosis (ABPA) should also be considered. Aspergillus sensitization and ABPA are not synonymous: sensitization indicates evidence of IgE-mediated reactivity to *Aspergillus*, whereas ABPA requires a broader clinicobiological and radiological assessment according to established diagnostic criteria. The 2024 revised ISHAM-ABPA guidelines provide updated recommendations for screening, diagnosis, classification, and management of ABPA and allergic bronchopulmonary mycosis [46].

The coexistence of both disorders also has important therapeutic implications. Asthma-directed anti-inflammatory treatment is appropriate when concomitant asthma is objectively supported, whereas management of bronchiectasis may require regular airway-clearance therapy, microbiological surveillance, and prompt treatment of exacerbations [1,3,6,33,34]. Because pediatric disease differs from adult asthma–bronchiectasis overlap with respect to etiology, airway growth and development, potential structural reversibility, infection patterns, and long-term therapeutic objectives, adult phenotyping models should be extrapolated to pediatric populations with caution [1,2,4,9,33].

Accordingly, this review uses the descriptive term “children with asthma and bronchiectasis” rather than implying the existence of a well-established pediatric overlap phenotype. Whether this coexistence represents comorbidity, a mechanistically linked continuum, or a distinct clinical entity remains unresolved. Nevertheless, recognizing the interaction between the two disorders is clinically relevant for integrating inflammatory control, mucus management, infection prevention, and pulmonary rehabilitation [4,5,6,7,8,9].

## 4. A Potential Biological Bridge: From Type 2 Inflammation to Structural Airway Damage

Asthma and bronchiectasis have traditionally been associated with distinct inflammatory patterns. Pediatric asthma, particularly allergic and eosinophilic phenotypes, is frequently characterized by Type 2 (T2) inflammation involving IL-4, IL-5, and IL-13, together with eosinophilic inflammation, IgE production, and epithelial-derived mediators [3,12]. Bronchiectasis, in contrast, is commonly associated with neutrophilic inflammation, recurrent infection, impaired mucociliary clearance, and structural airway injury [14,15,21,22]. Increasing evidence, however, indicates that bronchiectasis is biologically heterogeneous and may also include eosinophilic or mixed inflammatory profiles [13,14,15]. In asthma, IL-4 and IL-13 promote IgE synthesis, epithelial activation, goblet-cell metaplasia, and mucus hypersecretion, whereas IL-5 supports eosinophil maturation, recruitment, and survival [3,12,16,19]. Biomarkers such as blood eosinophil counts, fractional exhaled nitric oxide (FeNO), and serum IgE may help characterize T2-high inflammation, although they reflect partly distinct biological processes and should not be considered interchangeable [3,12]. The potential relevance of T2 inflammation in bronchiectasis has received increasing attention, particularly in adult studies identifying eosinophilic and mixed inflammatory endotypes [13,14,15]. Whether similar patterns are equally prevalent or clinically relevant in children remains uncertain. In children with coexisting asthma and bronchiectasis, T2 inflammation may coexist with infection-associated neutrophilic inflammation rather than replace it, resulting in a potentially mixed inflammatory environment.

Recurrent or persistent airway infection may further reshape this inflammatory environment by promoting non-Type 2 immune responses, including Th1- and Th17-associated pathways involved in antimicrobial host defense. Consequently, a child with an initially T2-high, allergic or eosinophilic asthma phenotype may develop a more complex mixed inflammatory profile once chronic infection and bronchiectatic airway disease become established. This should be viewed as inflammatory modification of the underlying asthma phenotype rather than evidence for a distinct asthma–bronchiectasis phenotype [14,15].

**Figure 1:**
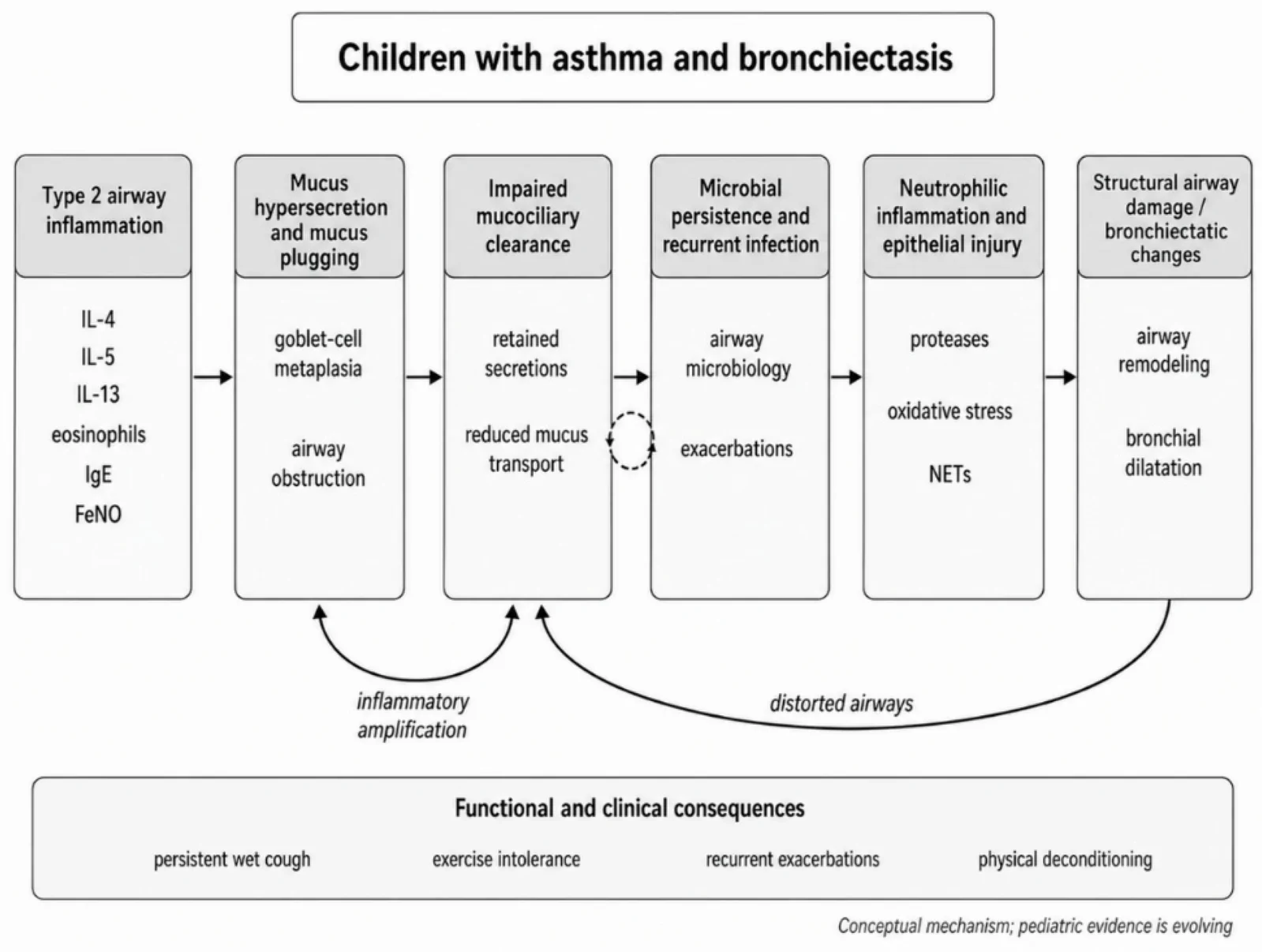
Proposed mechanistic pathway linking Type 2 inflammation, mucus dysfunction, impaired mucociliary clearance, infection, and structural airway damage in children with asthma and bronchiectasis. Arrows indicate proposed mechanistic interactions between the pathways and do not necessarily imply established causal relationships.

A biologically plausible link between T2 inflammation and structural airway injury is mucus dysfunction. IL-13-driven goblet-cell metaplasia and mucus hypersecretion may increase airway mucus burden and promote mucus plugging, thereby contributing to airflow obstruction and impaired mucociliary clearance [16,17,18,19]. Recurrent or persistent secretion retention may, in turn, favor microbial persistence and recurrent infection, promoting secondary neutrophilic inflammation and potentially reinforcing structural airway injury [14,15,22]. These proposed interactions are illustrated in Figure 1.

Importantly, current evidence does not support a simple causal progression from T2-high asthma to bronchiectasis in children. Bronchiectasis may precede asthma, arise from an independent underlying disorder, or develop through multiple interacting mechanisms [1,2,4,5]. Eosinophilic and neutrophilic inflammatory pathways may coexist and vary over time in relation to infection, exacerbations, corticosteroid exposure, allergen exposure, and changes in the airway microbiome [13,14,15].

The emerging model is therefore one of interacting inflammatory, mucociliary, and infectious mechanisms rather than a linear disease sequence. Identifying the predominant mechanisms in an individual child may help determine the relative emphasis placed on anti-inflammatory treatment, airway-clearance strategies, infection management, and pulmonary rehabilitation [14,47,48].

## 5. Mucus Hypersecretion and Mucociliary Dysfunction

Mucociliary clearance depends on coordinated ciliary activity, adequate mucus hydration and rheological properties, epithelial integrity, and sufficient expiratory airflow [14,16,19,21]. When these mechanisms are impaired, secretion retention becomes clinically relevant and may contribute to persistent respiratory symptoms and recurrent infection [14,21,22].

In T2-high asthma, IL-13-driven goblet-cell metaplasia and increased mucin production may promote mucus plugging and airflow obstruction [16,17,18,19]. In bronchiectasis, bronchial dilatation and distortion favor secretion pooling and impair mucociliary transport [1,2,21]. When both conditions coexist, asthma-related mucus hypersecretion and bronchoconstriction may further impair secretion mobilization [5,9,14].

Asthma control and effective airway clearance are related but distinct therapeutic goals. A child may have well-controlled asthma yet persistent wet cough or secretion retention; conversely, persistent cough should not automatically be interpreted as uncontrolled asthma. Cough characteristics, sputum burden, exacerbation pattern, microbiological findings, and response to airway-clearance interventions may help identify the predominant mechanism [1,20,33].

Mucociliary dysfunction is therefore an important therapeutic target. Airway-clearance techniques should be individualized according to age, developmental capacity, secretion burden, airflow obstruction, bronchial hyperresponsiveness, patient preference, family support, and clinical stability [1,24,33,34,35,38,40], with treatment intensity adjusted during exacerbations or changes in clinical status [1,34,49].

Adequate hydration and regular physical activity may support airway clearance. Hypertonic saline may facilitate expectoration but can induce bronchoconstriction in susceptible children, particularly those with concomitant asthma [36]. Exercise may enhance mucus mobilization but should complement, not replace, structured airway-clearance therapy when secretion burden is significant [24,35,39].

## 6. Infection, Inflammation, and the Vicious Vortex

The “vicious vortex” model emphasizes the reciprocal interaction between infection, inflammation, impaired clearance, and structural airway injury in bronchiectasis [14,21,22]. Persistent or recurrent lower-airway infection may promote neutrophilic inflammation and epithelial injury, while in children with coexisting asthma this process may coexist with Type 2/eosinophilic inflammation, resulting in a mixed and dynamic inflammatory profile [12,13,14,15,22].

This overlap complicates interpretation of cough, wheeze, dyspnea, and recurrent exacerbations, which may reflect uncontrolled asthma, secretion retention, infection, structural airway abnormalities, or combinations of these mechanisms [1,5,9,14]. Management should therefore avoid attributing persistent symptoms to a single pathway or disease component.

Exacerbations may intensify the vicious vortex. Increased secretion burden, cough, dyspnea, fatigue, and reduced activity can further impair clearance and functional status [14,21,49]. Rehabilitation should be adapted accordingly: stable periods emphasize effective airway clearance and physical activity, whereas exacerbations may require intensified airway-clearance therapy, closer symptom monitoring, and temporary adjustment of exercise intensity [1,24,34,49].

Bronchial hyperresponsiveness may also reduce tolerance of vigorous coughing, hyperosmolar inhaled therapies, and exercise [3,29,30,31,36]. Rehabilitation strategies should therefore be coordinated with asthma management and adapted to bronchial reactivity, secretion burden, clinical stability, and infection-related priorities.

## 7. Functional Consequences

The clinical impact of coexisting asthma and bronchiectasis extends beyond airway inflammation and structural abnormalities, affecting physical activity, exercise tolerance, school and recreational participation, and health-related quality of life [23,24,25,27,28,39]. Exercise limitation is frequently multifactorial and may reflect varying contributions from ventilatory limitation, exercise-induced bronchoconstriction, mucus-related airflow obstruction, respiratory muscle dysfunction, and physical deconditioning [25,27,28,29,30,31].

Reduced physical activity may itself worsen functional decline. Recurrent cough, dyspnea, fatigue, previous exercise-related symptoms, parental concern, and activity restriction may reinforce avoidance behavior and progressive aerobic and peripheral muscle deconditioning [23,27,28,31].

Respiratory muscle dysfunction may be an additional modifiable contributor. Limited pediatric evidence suggests that targeted respiratory muscle training may improve strength and selected functional outcomes in bronchiectasis, although its role in concomitant asthma remains uncertain [26].

Functional assessment should therefore extend beyond resting spirometry. Field exercise tests, cardiopulmonary exercise testing, habitual physical-activity assessment, respiratory muscle strength measurements, and patient-reported outcomes may help identify predominant contributors and guide rehabilitation priorities [23,25,26,27,28,29,30,31,32].

## 8. Why Pulmonary Rehabilitation Should Be Individualized

Pulmonary rehabilitation should be individualized because the relative contributions of mucus retention, bronchial hyperresponsiveness, exercise-induced bronchoconstriction, physical deconditioning, respiratory muscle dysfunction, recurrent exacerbations, and treatment burden vary among children and over time [3,24,25,26,29,30,31,40,47,48]. Rehabilitation priorities should therefore be guided by the predominant functional and physiological limitations rather than by diagnosis alone [47,48].

Individualization should also incorporate developmental and practical considerations. Age, developmental stage, ability to cooperate, family involvement, treatment burden, school routines, preferences, and adherence influence feasibility and effectiveness [1,24,33,34,38,40,47,48]. Younger children may require play-based, caregiver-supported approaches, whereas adolescents may benefit from greater autonomy and integration into preferred activities [39,40].

Individualized pulmonary rehabilitation does not replace disease-specific treatment. Optimal asthma management, prevention and treatment of respiratory infections, and evaluation of underlying causes of bronchiectasis remain essential [1,3,33,34,38]. Rehabilitation should address residual functional and physiological limitations within coordinated multidisciplinary care [24,40,47,48].

At present, Type 2 biomarker status should primarily inform disease-specific asthma management rather than the direct selection of a particular airway-clearance technique or exercise modality. There is currently no evidence that blood eosinophils, FeNO, serum IgE, or other Type 2 biomarkers should determine the choice of ACT, exercise format, or respiratory muscle training strategy in children with coexisting asthma and bronchiectasis. Their relevance to rehabilitation is therefore indirect, through optimization of asthma control, reduction of bronchial hyperresponsiveness and exacerbation burden, and improved ability to participate in rehabilitation.

The added value of the present framework is not the introduction of entirely new rehabilitation modalities, but the integration of existing pediatric bronchiectasis rehabilitation principles with asthma-specific considerations in children in whom both conditions coexist. Compared with bronchiectasis-focused consensus recommendations and treatable-traits approaches, the proposed framework explicitly incorporates bronchial hyperresponsiveness, exercise-induced bronchoconstriction, asthma control, Type 2 inflammatory assessment, and the need to distinguish mucus-related symptoms from asthma-related symptoms when selecting and sequencing rehabilitation interventions. It therefore aims to translate established rehabilitation components into a practical, mechanism-informed clinical framework for this specific and insufficiently studied population [40,47,48].

Pulmonary rehabilitation can therefore be conceptualized as an iterative process of assessment, identification of functional limitations, targeted intervention, outcome measurement, reassessment, and adaptation [32,47,48].

## 9. Airway-Clearance Techniques

Airway-clearance techniques (ACTs) are a core component of pediatric bronchiectasis management because secretion retention contributes to chronic cough, recurrent infection, airway inflammation, and exacerbations [1,24,33,34,35,38]. In coexisting asthma, selection should also consider bronchial hyperresponsiveness, variable airflow obstruction, and treatment-related symptom provocation [3,29,30,31,36].

ACTs mobilize secretions toward the central airways to facilitate expectoration or swallowing through controlled breathing, lung-volume modulation, positive expiratory pressure, oscillation, forced expiration, or postural effects [35,38]. No single technique is consistently superior; selection should be individualized according to age, developmental capacity, secretion burden, airflow limitation, bronchial reactivity, clinical stability, tolerability, and preferences [1,24,33,34,35,38,40]. The principal techniques and asthma-specific considerations are summarized in Table 2.

Active cycle of breathing techniques, PEP/OPEP therapy, autogenic or modified autogenic drainage, postural drainage, and forced expiratory techniques may all be considered according to age, developmental capacity, secretion burden, and physiological needs [1,24,33,34,35,38]. Younger children may require therapist- or caregiver-assisted approaches; in practice, correct technique, tolerability, and adherence may matter more than theoretical differences between methods [24,35,38,40].

In a randomized pediatric trial, Zeren et al. evaluated whether adding oscillatory positive expiratory pressure (OPEP) to a conventional chest physiotherapy program provided additional benefit in children with bronchiectasis. The addition of OPEP did not result in a clear additional improvement in exercise capacity or spirometric outcomes compared with physiotherapy alone, although treatment adherence was improved. These findings suggest that OPEP may offer practical advantages for some children, but they do not establish superior physiological effectiveness over a structured physiotherapy program [37].

Coexisting asthma may influence ACT tolerability. Forced expiratory maneuvers, repeated coughing, hyperosmolar inhaled therapies, or increased ventilatory demand may provoke symptoms in susceptible children [3,29,30,31,36]. ACT sessions should therefore be coordinated with asthma management when appropriate, although pediatric evidence on optimal bronchodilator-ACT sequencing remains limited.

ACT frequency and intensity should reflect clinical status. Stable periods require a sustainable, age-appropriate routine, whereas exacerbations may require more frequent or modified sessions without imposing a treatment burden that undermines adherence [1,24,34,49].

## 10. Exercise Training and Physical Reconditioning

Exercise training is an important component of pulmonary rehabilitation because physical inactivity and deconditioning contribute to dyspnea, fatigue, and functional limitation [23,25,27,28,39,41,42]. In children with coexisting asthma and bronchiectasis, exercise may improve cardiorespiratory fitness, confidence, and age-appropriate participation in daily, school, and recreational activities [24,27,28,39].

Jones et al. randomized 47 children with non-cystic fibrosis bronchiectasis to a play-based therapeutic exercise program or usual care. The primary outcome, remaining exacerbation-free over 12 months, did not differ significantly between groups (OR 1.51, 95% CI 0.32–7.5). Although physical activity increased immediately after the intervention, this improvement was not maintained at follow-up. These findings support the feasibility of structured exercise in children with bronchiectasis but do not demonstrate a durable reduction in exacerbations or sustained long-term improvement in physical activity [39].

Aerobic, resistance, and interval training may be selected according to baseline fitness, symptoms, developmental stage, goals, and preferences [27,28,31,39,41,42]. Intensity and duration should progress gradually according to tolerance. Interval training may be useful in marked deconditioning or when prolonged continuous exercise provokes limiting symptoms [27,31,42]. The principal exercise and respiratory muscle training modalities are summarized in Table 3.

Coexisting asthma requires particular attention because exercise-induced bronchoconstriction (EIB) may reduce exercise tolerance and reinforce activity avoidance [3,29,30,31]. Children with exercise-related respiratory symptoms should be assessed for asthma control and, when indicated, EIB before substantial progression of intensity. Warm-up strategies, environmental adaptation, symptom monitoring, and coordination with asthma management may improve tolerability and safety [3,29,30,31].

Training intensity should be individualized using objective exercise assessment where available or practical measures such as heart rate, perceived exertion, symptom response, and field-test performance [25,27,28,29,30,31,41,42]. Long-term success depends on integrating physical activity into preferred recreational activities, school participation, and family routines [27,28,39].

Exercise prescription should reflect predominant limitations: progressive aerobic and resistance training for deconditioning, integration with airway-clearance strategies when secretion retention is significant, and adaptation of intensity, warm-up, environmental exposure, and treatment timing when bronchial hyperresponsiveness or EIB is present [24,27,28,29,30,31,39,47,48].

## 11. Respiratory Muscle Training

Respiratory muscle training (RMT) may be a useful adjunct when respiratory muscle weakness, clinically significant fatigue, or reduced exercise tolerance is present, but current pediatric evidence does not support routine use in all children with coexisting asthma and bronchiectasis [26,40,42].

Inspiratory muscle training (IMT), the most studied approach, uses repeated inspiratory efforts against external resistance with progressive loading relative to maximal inspiratory pressure (MIP) [26,42]. Limited pediatric bronchiectasis data suggest potential improvements in respiratory muscle strength and selected functional or pulmonary-function outcomes, but the available evidence is based on small and methodologically limited studies [26].

Chen et al. studied 17 children with bronchiectasis who completed a 24-week incremental-load respiratory muscle training program. Improvements were reported in respiratory muscle strength and selected pulmonary-function measures; however, the study did not include a parallel control group. Consequently, the observed changes cannot be attributed confidently to respiratory muscle training alone, because concurrent treatment, normal growth and development, and repeated testing may also have contributed. These methodological limitations substantially reduce the certainty of the evidence supporting respiratory muscle training in this population [26].

Respiratory muscle function should ideally be assessed before targeted training. MIP and maximal expiratory pressure (MEP) may identify clinically relevant weakness when age-appropriate reference values and standardized techniques are used [26,42]. RMT may be considered when an objective deficit is documented or when respiratory muscle dysfunction plausibly contributes to dyspnea, fatigue, or exercise limitation [26,40].

RMT should preferably be undertaken when asthma is clinically stable and coordinated with asthma management when bronchial hyperresponsiveness or exercise-related symptoms are present [3,29,30,31]. Age, developmental capacity, cooperation, device simplicity, family support, and treatment burden also influence feasibility and adherence [40,47].

RMT should be regarded as a targeted adjunct and should not replace aerobic conditioning, airway-clearance therapy, or disease-specific treatment [26,40,42]. The principal intervention studies informing the proposed rehabilitation framework are summarized in Table 4.

No identified intervention study specifically evaluated children with both confirmed asthma and bronchiectasis; therefore, the proposed framework integrates evidence from pediatric bronchiectasis, pediatric asthma, and selected adult studies.

## 12. Exercise-Induced Bronchoconstriction During Pulmonary Rehabilitation

Exercise-induced bronchoconstriction (EIB) is an important consideration during pulmonary rehabilitation [3,29,30,31]. Cough, wheeze, chest tightness, dyspnea, or reduced performance are not specific to EIB and may also reflect mucus retention, deconditioning, dysfunctional breathing, or exercise-induced laryngeal obstruction [29,30].

Misattributing exertional symptoms to uncontrolled asthma may lead to unnecessary treatment escalation, whereas unrecognized EIB may reinforce activity avoidance [29,30,31]. Normal baseline spirometry does not exclude EIB; objective bronchoprovocation or exercise testing should be considered when the diagnosis remains uncertain and would influence management [3,29,30].

When EIB is confirmed, pulmonary rehabilitation should be coordinated with asthma management rather than restricted. Optimized asthma control, appropriate warm-up, environmental adaptation, and guideline-directed pre-exercise medication when indicated may improve exercise tolerance and sustain physical activity [3,29,30,31].

In bronchiectasis, exercise-related cough may reflect secretion mobilization rather than bronchoconstriction. Symptom timing, objective lung-function changes, sputum production, associated wheeze or chest tightness, and bronchodilator response may help distinguish EIB from mucus-related symptoms or airflow limitation [1,24,25].

Airway clearance and exercise may require individualized sequencing: some children benefit from clearance before exercise, whereas others mobilize secretions during activity and perform ACTs afterward [24,39,40]. Evidence defining the optimal sequence of bronchodilator therapy, ACTs, and exercise in this population remains limited.

EIB should therefore be viewed as a potentially modifiable barrier, not a contraindication to exercise, with rehabilitation focused on safe and sustained age-appropriate physical activity [29,30,31].

## 13. A Proposed Multidisciplinary Clinical Framework for Pulmonary Rehabilitation

Children with coexisting asthma and bronchiectasis require coordinated disease control and functional rehabilitation. Because inflammation, bronchial hyperresponsiveness, mucus retention, recurrent infection, exercise limitation, respiratory muscle dysfunction, and deconditioning contribute in varying proportions, pulmonary rehabilitation should begin with structured multidimensional assessment [1,3,24,25,26,29,30,31,33,34,40,47,48].

The first step is to confirm clinical stability and identify predominant clinical and functional limitations. Assessment should include asthma control, recent exacerbations, secretion burden and wet-cough characteristics, relevant microbiology, exercise-related symptoms, habitual physical activity, functional exercise capacity, respiratory muscle performance when indicated, and barriers to adherence [1,3,24,25,26,29,30,31,32,33,34,40,47,48,49].

Functional assessment should be age-appropriate and selected according to the child’s developmental level, symptoms, and clinical question. Field exercise tests such as the 6-min walk test may be used to assess functional exercise capacity, while cardiopulmonary exercise testing can provide more detailed physiological information when available and clinically indicated. Respiratory muscle weakness should be established objectively using maximal inspiratory and/or expiratory pressure measurements interpreted against age-appropriate reference values. Reassessment should focus on changes in exercise tolerance, symptom burden, secretion clearance, respiratory muscle performance, exacerbation-related recovery, participation, and patient- or caregiver-reported outcomes, rather than on spirometry alone.

The second step is to identify rehabilitation priorities across five practical domains: (1) mucus retention and impaired airway clearance; (2) bronchial hyperresponsiveness and/or EIB; (3) reduced exercise capacity and deconditioning; (4) respiratory muscle dysfunction; and (5) exacerbation-related functional decline [24,25,26,29,30,31,40,47,48]. These domains may coexist and change over time [47,48].

**Figure 2:**
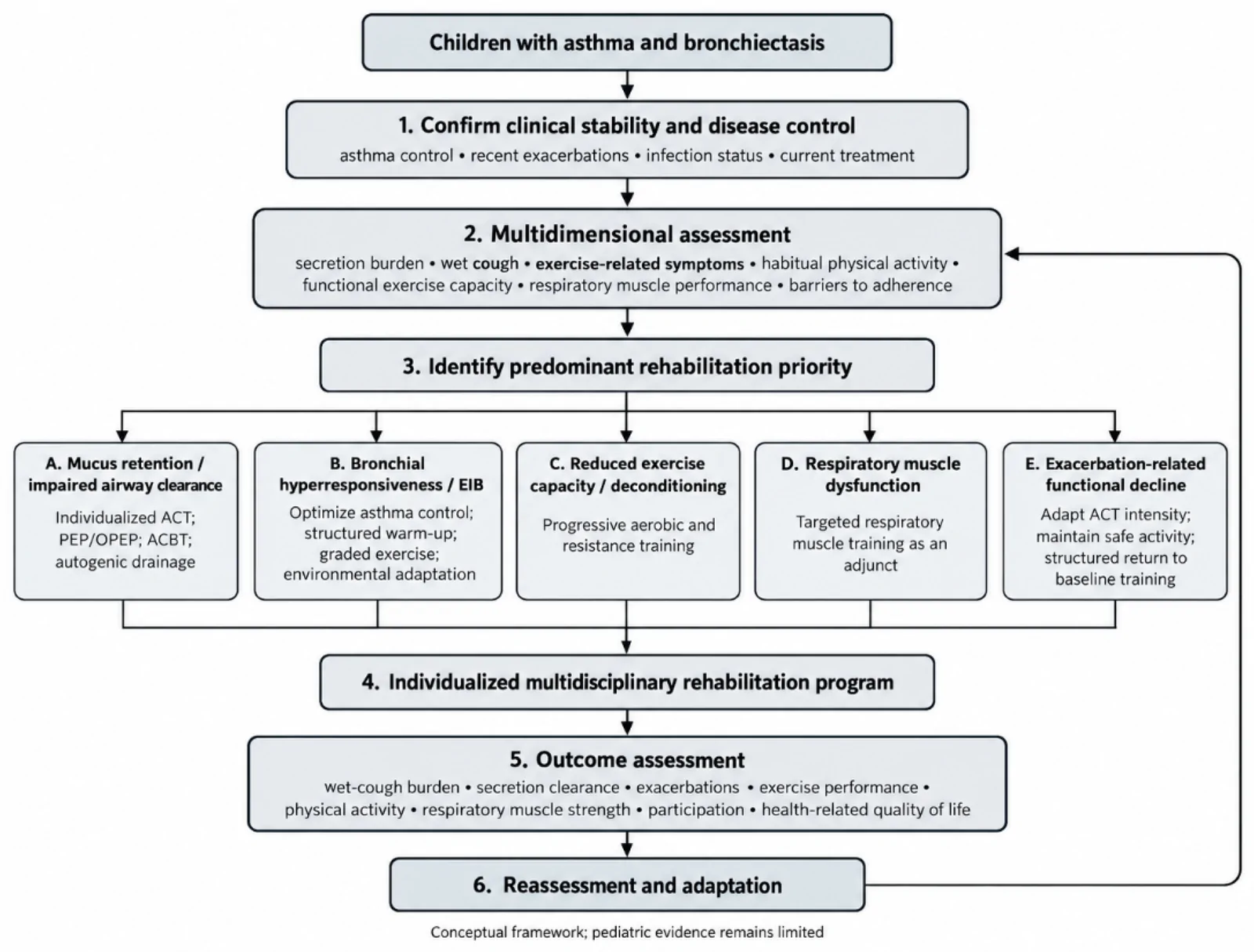
Clinical decision-making framework for individualized pulmonary rehabilitation in children with asthma and bronchiectasis.

Rehabilitation sessions should be adapted or temporarily interrupted when clinically relevant symptom deterioration occurs, including new or worsening wheeze, chest tightness, disproportionate dyspnea, marked fatigue, dizziness, inability to recover appropriately after exercise, or features suggestive of an acute respiratory exacerbation. A sudden increase in wet cough or secretion burden, fever, or other signs of infection should prompt reassessment of clinical stability before continuing or progressing the program. Similarly, persistent exercise-related symptoms despite optimized asthma management should prompt reconsideration of bronchial hyperresponsiveness or exercise-induced bronchoconstriction before further intensity progression. These decisions should be individualized and based on clinical judgment, because pediatric evidence defining uniform interruption thresholds in children with coexisting asthma and bronchiectasis is lacking. A practical clinical decision-making pathway integrating these domains is illustrated in Figure 2.

For example, a child with stable asthma but persistent wet cough, daily sputum production, and reduced exercise tolerance may initially require prioritization of airway-clearance therapy and graded physical reconditioning. By contrast, a child with minimal secretion burden but exertional wheeze and a documented fall in lung function after exercise would require optimization of asthma control and exercise-induced bronchoconstriction management before progression of exercise intensity. These examples are illustrative and are intended to demonstrate how the dominant clinical limitation may guide rehabilitation priorities rather than to prescribe a fixed treatment sequence.

The proposed mechanism-informed, multidisciplinary rehabilitation framework is summarized in Table 5.

Interventions should match the predominant priority: individualized airway-clearance therapy for mucus retention [1,24,33,34,35,36,37,38,40]; optimization of asthma control and exercise tolerance when bronchial hyperresponsiveness or EIB limits participation [3,29,30,31]; progressive aerobic and resistance training for deconditioning [27,28,39,41,42]; selective RMT for documented respiratory muscle impairment [26,40]; and flexible adaptation of airway-clearance intensity and exercise goals during recurrent exacerbations [1,24,34,49].

A multidisciplinary approach is central because rehabilitation priorities span clinical and functional domains. Within a rehabilitation-centered model, the Physical and Rehabilitation Medicine (PRM) physician may coordinate functional assessment, individualized goals, interventions, and outcome monitoring. Pediatric pulmonologists and allergists provide disease-specific management, physiotherapists implement and adapt rehabilitation interventions, and nurses, psychologists, dietitians, and other professionals contribute according to individual needs [1,24,33,34,40,47,48,50].

Pulmonary rehabilitation should follow an iterative process of assessment, targeted intervention, outcome measurement, reassessment, and adaptation [32,47,48]. Treatment success should be evaluated using both objective functional measures and patient-important outcomes. Relevant measures may include functional exercise capacity assessed by field walking tests or cardiopulmonary exercise testing where available, habitual physical activity, respiratory muscle strength, pulmonary function when clinically informative, wet-cough and secretion burden, exacerbation frequency, recovery time after exacerbations, dyspnea and exercise-related symptoms, school and sports participation, adherence, treatment burden, and health-related quality of life [23,24,25,26,27,28,32,49]. In children, patient- and caregiver-reported outcomes may provide additional information on daily functioning, symptom perception, and the practical impact of rehabilitation. For example, the Pediatric Asthma Quality of Life Questionnaire (PAQLQ) may be used in school-aged children and adolescents with asthma to assess the impact of symptoms, activity limitation, and emotional function. Selection of patient- or caregiver-reported outcome measures should be adapted to the child’s age, clinical condition, and purpose of assessment.

The same diagnostic combination may therefore require different strategies. T2-high asthma with EIB and minimal secretion burden may primarily require optimized asthma management and graded exercise [3,12,29,30,31], whereas frequent wet cough, substantial mucus retention, and recurrent infective exacerbations may justify greater emphasis on airway-clearance therapy before progressive conditioning [1,24,33,34,35,36,37,38,49].

In children with severe asthma and a pronounced Type 2 inflammatory phenotype, biologic therapy may represent an important complementary component of individualized management. Targeted biologic agents should not be considered a pulmonary rehabilitation intervention per se, but improved control of Type 2 inflammation may reduce exacerbation burden, bronchial hyperresponsiveness, and symptom limitation, thereby facilitating participation in exercise training and other rehabilitation components. Selection of biologic therapy should remain based on established pediatric severe-asthma criteria, including clinical phenotype, biomarkers, exacerbation history, and previous treatment response [3]. Importantly, evidence specifically examining the interaction between biologic therapy and pulmonary rehabilitation in children with coexisting asthma and bronchiectasis is currently lacking, and this relationship warrants prospective investigation.

This framework is evidence-informed and conceptual rather than a validated pediatric treatment guideline; prospective studies are needed to determine whether it improves patient-centered outcomes.

## 14. Evidence Gaps and Future Directions

Evidence specifically addressing children with coexisting asthma and bronchiectasis remains limited because most pediatric studies evaluate the conditions separately [1,10,11,24,33,47,48].

A major priority is to clarify which children are at greatest risk of bronchiectatic changes and whether persistent Type 2 inflammation, mucus plugging, infection, airway remodeling, or their interaction contributes to structural progression [10,11,12,13,14,15,16,17,18,19]. Longitudinal pediatric studies integrating biomarkers, imaging, airway microbiology, lung function, exacerbation burden, and functional outcomes are needed to define disease trajectories and predictors [10,11,32,47,48].

Optimal sequencing of pharmacological therapy and rehabilitation also remains uncertain. Pediatric studies should assess bronchodilator timing relative to airway-clearance therapy, sequencing of exercise and ACTs, and adaptation of these interventions during acute exacerbations [24,29,30,31,35,36,37,38,39,40].

Comparative evidence for individual ACTs remains limited. Future studies should examine differential responses according to secretion burden, airflow obstruction, bronchial hyperresponsiveness, age, developmental capacity, tolerability, and adherence. Exercise and respiratory muscle training also require greater standardization of intensity, duration, progression, patient selection, and clinically meaningful outcomes [25,26,27,28,29,30,31,39,41,42].

Validated tools for rehabilitation stratification are also lacking. A pediatric framework incorporating secretion burden, asthma control, exercise tolerance, respiratory muscle function, exacerbation history, bronchial hyperresponsiveness, functional status, and adherence may help identify predominant priorities but requires prospective validation [32,47,48].

Future studies should prioritize patient-centered outcomes, including physical activity, school and sports participation, treatment burden, and health-related quality of life, rather than predominantly spirometric or laboratory measures [23,24,27,28,32,39]. Telerehabilitation, wearable monitoring, and remote adherence support may facilitate individualized care, but pediatric evidence remains limited.

Future research should move toward prospective, mechanism-informed, function-oriented, and patient-centered rehabilitation studies [47,48]. In particular, multicenter prospective pediatric cohorts and randomized controlled trials are needed to validate the proposed individualized rehabilitation framework across different clinical phenotypes, age groups, and healthcare settings. Such studies should use standardized rehabilitation protocols, predefined functional and patient-important outcome measures, and longitudinal follow-up to assess feasibility, effectiveness, reproducibility, and generalizability. Multicenter designs will be especially important to determine whether individualized pulmonary rehabilitation provides clinically meaningful benefits beyond standardized approaches and to establish which patient subgroups are most likely to benefit.

## 15. Conclusions

Children with coexisting asthma and bronchiectasis are clinically heterogeneous, with variable contributions from airway inflammation, bronchial hyperresponsiveness, mucus retention, impaired mucociliary clearance, recurrent infection, exercise limitation, respiratory muscle dysfunction, and deconditioning [1,2,4,5,6,7,8,9,14,24,25,26,27,28,29,30,31,40,47,48].

Current evidence supports airway-clearance therapy and regular physical activity in pediatric bronchiectasis, together with optimal control of asthma-related inflammation and bronchial hyperresponsiveness [1,3,24,27,28,29,30,31,33,34,35,36,37,38,39]. Direct evidence on pulmonary rehabilitation in children with both conditions remains limited [10,11,24,47,48].

Pulmonary rehabilitation should therefore be individualized by identifying predominant clinical, physiological, and functional limitations and tailoring interventions accordingly [24,26,40,47,48].

Disease control and functional recovery are complementary but distinct goals. The proposed framework integrates inflammatory control, secretion management, exercise capacity, respiratory muscle function, and age-appropriate participation within coordinated multidisciplinary care [24,26,32,40,47,48].

Because chronic airway inflammation and recurrent infection can impair mucociliary clearance and promote secretion retention, systematic identification and treatment of impaired airway clearance represent an important component of comprehensive management. The proposed rehabilitation framework therefore provides a practical approach for tailoring secretion-clearance strategies to the individual child while integrating inflammatory control, infection management, and functional rehabilitation.

Given current evidence limitations, this approach should be regarded as an evidence-informed conceptual framework and should not be interpreted as a prospectively validated pediatric treatment pathway. Prospective studies are needed to determine whether mechanism-informed, function-oriented, and patient-centered rehabilitation improves clinically meaningful outcomes.

## Figures and Tables

**Table 1:** Key Pediatric Evidence on Asthma and Bronchiectasis.

Study	Population	Main Findings	Relevance
Lo et al., 2019 [11]	30 children with severe asthma; retrospective CT study	Bronchiectasis in 27%; bronchial wall thickening in 80%; air trapping in 60%	Demonstrates that bronchiectatic changes can coexist with severe pediatric asthma
van den Bosch et al., 2024 [10]	131 children with severe asthma; retrospective CT analysis	Bronchiectasis in 28% by manual assessment; bronchial wall thickening in 88.8%	Confirms frequent structural airway abnormalities in severe pediatric asthma
Chang et al., 2021 [1]	ERS pediatric bronchiectasis guideline	Defines pediatric bronchiectasis; emphasizes etiological work-up, airway-clearance therapy, and potential reversibility	Provides the pediatric framework for interpreting bronchiectasis in children with asthma
Chang et al., 2021 [33]	ERS guideline summary	Highlights pediatric-specific etiology, microbiology, prognosis, and treatment principles	Supports cautious extrapolation from adult asthma–bronchiectasis data

Note: Frequencies of bronchiectatic or structural airway changes reported in CT-based studies derive from selected referral, tertiary-care, or severe-asthma cohorts and should not be interpreted as prevalence estimates for the general pediatric asthma population. Observational cohort data are presented separately from guideline- or review-based evidence to reflect differences in study design and level of inference.

**Table 2:** Airway-Clearance Techniques in Children with Bronchiectasis and Considerations in Concomitant Asthma.

Technique	Main Mechanism	Potential Clinical Role	Asthma-Specific Considerations
ACBT	Breathing control, thoracic expansion, forced expiration	Older children and adolescents able to cooperate	Forced expiratory maneuvers may provoke cough or bronchospasm in hyperresponsive airways
PEP	Maintains airway patency and promotes secretion movement	Mucus retention with expiratory flow limitation	Resistance should be individualized; monitor tolerance
OPEP	Combines PEP with oscillations to mobilize mucus	Viscous secretions and difficult expectoration	May provoke intense coughing; added benefit over standard ACTs remains uncertain
Autogenic drainage	Controlled breathing at different lung volumes	Cooperative older children/adolescents	Requires concentration and good respiratory control
Modified autogenic drainage	Therapist-assisted adaptation of autogenic drainage	Younger children	Depends on therapist expertise and caregiver involvement
Postural drainage ± manual techniques	Gravity and manual mobilization of secretions	Younger children or those unable to perform active techniques	Head-down positions may be poorly tolerated; caution with reflux or respiratory distress
Huff/forced expiratory technique	Increases expiratory airflow and moves central secretions	Central secretion mobilization	Intensity should be adapted in airflow limitation or bronchial hyperresponsiveness
Exercise as adjunct	Increases ventilation and expiratory airflow	Mucus retention plus deconditioning	EIB should be identified and controlled; exercise does not replace ACTs
Hypertonic saline + ACT	Improves airway-surface hydration and expectoration	Selected children with thick secretions	May induce bronchoconstriction; tolerance should be assessed carefully

Abbreviations: ACBT, active cycle of breathing techniques; ACTs, airway-clearance techniques; EIB, exercise-induced bronchoconstriction; OPEP, oscillating positive expiratory pressure; PEP, positive expiratory pressure.

**Table 3:** Exercise and Respiratory Muscle Training in Children with Asthma and Bronchiectasis.

Intervention	Main Objective	Potential Outcomes	Key Considerations
Aerobic training	Improve cardiorespiratory fitness	Exercise capacity, dyspnea, QoL, participation	Progressive intensity; assess asthma control and EIB
Interval training	Reduce sustained ventilatory stress	Exercise tolerance, adherence	May be useful in EIB or marked deconditioning
Resistance training	Improve peripheral muscle strength	Functional capacity, participation	Adapt to age and developmental stage
Exercise + ACT	Combine conditioning with secretion mobilization	Airway clearance, exercise capacity	Optimal sequencing remains uncertain
Inspiratory muscle training	Improve inspiratory muscle strength/endurance	MIP, dyspnea, exercise capacity	Consider when objectively documented weakness is present
Expiratory muscle training	Improve expiratory muscle performance	MEP, cough effectiveness	Pediatric evidence remains limited
Structured warm-up	Reduce exercise-related respiratory symptoms	Improved exercise tolerance	Particularly relevant in children with EIB
Return-to-activity program	Restore function after exacerbation	Physical activity, participation	Progress according to clinical recovery

Abbreviations: ACT, airway-clearance technique; EIB, exercise-induced bronchoconstriction; MEP, maximal expiratory pressure; MIP, maximal inspiratory pressure; QoL, quality of life.

**Table 4:** Principal Intervention Evidence Informing the Proposed Rehabilitation Framework.

Study	Population/Evidence Base	Intervention	Comparator	Duration/Follow-Up	Main Findings and Limitations
Zeren et al. [37]	42 children with non-CF bronchiectasis	OPEP + multicomponent chest physiotherapy	Multicomponent chest physiotherapy alone	8 weeks	No additional improvement in exercise capacity or spirometry with OPEP; adherence improved. Does not demonstrate superior physiological effectiveness of OPEP.
Jones et al. [39]	47 children with non-CF bronchiectasis	Play-based therapeutic exercise program	Waitlist control/usual care	8-week intervention; follow-up at 6 and 12 months	No significant difference in remaining exacerbation-free at 12 months (OR 1.51, 95% CI 0.32–7.5). Physical activity improved immediately after the program but was not maintained at follow-up.
Chen et al. [26]	17 children with bronchiectasis	Incremental-load respiratory muscle training	No parallel control group; pre–post comparison	24 weeks	Improvements in respiratory muscle strength and selected pulmonary-function measures; absence of a parallel control group limits causal attribution.
Jiang et al. [27]	Children with asthma; systematic review/network meta-analysis	Exercise rehabilitation	Multiple comparators across included studies	Variable across included studies	Supports potential functional benefits of exercise rehabilitation; heterogeneous interventions and not specific to bronchiectasis.
Jing et al. [28]	Children with asthma; systematic review/meta-analysis	Physical activity/exercise	Multiple comparators across included studies	Variable across included studies	Suggests benefits for selected lung-function and quality-of-life outcomes; heterogeneous evidence and not specific to bronchiectasis.
Lee et al. [41]	Adults with non-CF bronchiectasis	Exercise training	Multiple comparators across included studies	Variable across included studies	Provides supportive adult evidence for exercise training but represents indirect evidence for pediatric practice.

**Table 5:** Mechanism-Informed Individualization of Pulmonary Rehabilitation in Children with Asthma and Bronchiectasis.

Predominant Problem	Key Assessment	Main Rehabilitation Strategy	Main Outcomes to Monitor
Mucus retention/impaired airway clearance	Wet cough, secretion burden, sputum characteristics, previous ACT response	Individualized ACT; PEP/OPEP; ACBT; autogenic drainage; age-adapted manual techniques	Secretion clearance, wet-cough burden, exacerbations
Bronchial hyperresponsiveness/EIB	Asthma control, exercise-related symptoms, objective EIB assessment when feasible	Optimize asthma control; structured warm-up; graded exercise; environmental adaptation	Exercise symptoms, tolerance, activity participation
Physical deconditioning	Functional exercise capacity, habitual activity, fatigue, participation	Progressive aerobic and resistance training	Exercise capacity, physical activity, QoL
Respiratory muscle dysfunction	MIP/MEP or other objective measures when clinically indicated	Targeted respiratory muscle training as an adjunct	Respiratory muscle strength, dyspnea, exercise performance
Recurrent exacerbation-related decline	Exacerbation frequency, recovery pattern, functional loss	Adapt ACT intensity; maintain safe activity; structured return to baseline training	Recovery time, exacerbation burden, functional preservation
Multiple coexisting limitations	Integrated respiratory and functional assessment	Combined, prioritized multidisciplinary program with repeated reassessment	Patient-centered and disease-specific outcomes

Abbreviations: ACBT, active cycle of breathing techniques; ACT, airway-clearance technique; EIB, exercise-induced bronchoconstriction; MEP, maximal expiratory pressure; MIP, maximal inspiratory pressure; OPEP, oscillating positive expiratory pressure; PEP, positive expiratory pressure; QoL, quality of life.

## Data Availability

No new data were created or analyzed in this study. Data sharing is not applicable to this article. All information discussed in this review is derived from previously published studies cited in the manuscript.
